# *κ*-Carrageenan Enhances Lipopolysaccharide-Induced Interleukin-8 Secretion by Stimulating the Bcl10-NF-*κ*B Pathway in HT-29 Cells and Aggravates* C*.* freundii*-Induced Inflammation in Mice

**DOI:** 10.1155/2017/8634865

**Published:** 2017-01-09

**Authors:** Wei Wu, Zhanghe Zhen, Tingting Niu, Xiaojuan Zhu, Yuli Gao, Jiangyan Yan, Yu Chen, Xiaojun Yan, Haimin Chen

**Affiliations:** ^1^Key Laboratory of Applied Marine Biotechnology of Zhejiang Province, Ningbo University, Ningbo, Zhejiang 315211, China; ^2^Collaborative Innovation Center for Zhejiang Marine High-Efficiency and Healthy Aquaculture, Zhejiang 315211, China

## Abstract

*Background.* The dietary usage of carrageenan as common food additive has increased observably over the last 50 years. But there is substantial controversy about its safety.* Methods.* We investigated whether the *κ*-carrageenan could enhance lipopolysaccharide-induced IL-8 expression by studying its actions on the TLR4-NF-*κ*B pathway. The aggravating effect of *κ*-carrageenan on* Citrobacter freundii* DBS100-induced intestinal inflammation was also investigated in a mouse model.* Results.* Our data show that *κ*-carrageenan pretreatment promoted LPS-induced IL-8 expression in HT-29 cells. Although CD14, MD-2, and TLR4 were upregulated, the binding of LPS was not enhanced. However, the pathway of Bcl10-NF-*κ*B was triggered. Interestingly, *κ*-carrageenan competitively blocked the binding of FITC-LPS. Furthermore, pretreatment with *κ*-carrageenan for one week previous to gavage with* C. freundii* DBS100 markedly aggravated weight loss, mortality, and colonic damage. The secretion of cytokines was unbalanced and the ratio of Tregs was decreased significantly. In addition, *κ*-carrageenan, together with* C. freundii* DBS100, enhanced the transcription and secretion of TLR4 and NF-*κ*B.* Conclusions*. *κ*-Carrageenan can synergistically activate LPS-induced inflammatory through the Bcl10-NF-*κ*B pathway, as indicated by its aggravation of* C. freundii* DBS100-induced colitis in mice.* General Significance.* Our results suggest that *κ*-carrageenan serves as a potential inflammatory agent that magnifies existing intestinal inflammation.

## 1. Introduction

Carrageenan, a high molecular weight sulfated polysaccharide derived from a red seaweed [1–3], is used as a food additive to improve the formation of food products [4, 5]. Despite its wide application, the safety of carrageenan is controversial. Earlier work demonstrated that intake of carrageenan may cause anabasis or cancer of the gut in animal models [1, 6–11]. Moreover, carrageenan has been used to study mediators of inflammation [6, 9, 12]. On the other hand, some investigations have concluded that carrageenan is safe for humans [13–15]. Reports from many agencies emphasize that food grade carrageenan is safe to use as a “nonspecified” food additive [1, 2, 16, 17]. Therefore, the safety of carrageenan has remained highly controversial [1].

Extensive studies have been performed to examine carrageenan safety at the cell level. Carrageenan has been demonstrated to trigger innate immune responses through the interaction between an *α*-d-Gal-(1–3)-d-Gal epitope on carrageenan and the TLR4 [18–20]. The role of TLR4 in the innate immune response to carrageenan has also been demonstrated using a TLR4−/− mouse [21]. Furthermore, carrageenan has been indicated to activate NF-*κ*B and AP-1 pathways through TLR4 in macrophages, leading to the upregulation of tumor necrosis factor alpha (TNF-*α*) secretion [22]. However, in these studies, the degree of inflammation caused by carrageenan is low. For example, 10 *μ*g/mL *λ*-carrageenan only increases the IL-8 secretion level in NCM460 human colonic epithelial cells 4-fold, with even lower responses for *κ*- and ι-carrageenan [19]. By comparison, 1 ng/mL lipopolysaccharide (LPS) can induce >10-fold increase in IL-8 in the same cell line and can trigger a hundredfold upregulation for the TNF-*α* expression in macrophages [23, 24]. On the basis of these observations, investigators have pondered whether the inflammatory effect of carrageen is plenty to trigger severe inflammatory reactions in vivo [4].

In our previous studies, we found that epithelial monolayers composed of differentiated Caco-2 cells cocultured with THP-1 cells were easily damaged by carrageenan [25]. Because the intestinal tract is in constant contact with microorganism, including pathogens, the intestinal tract may undergo low levels of chronic inflammation, especially if pathogens accumulate. In this situation, the inflammation provoked by the pathogens might be enhanced by carrageenan. This situation may partially explain why carrageenan has been found to induce colonic inflammation in some studies, but not in others. Therefore, we considered the possibility that carrageenan may act as a conditional inflammatory agent. According to our hypothesis, when the gut is in an inflammatory state, carrageenan will collaboratively aggravate the colitis.

LPS is an important constituent of the external wall of gram-negative bacteria, and lots of the inflammatory signals were triggered through LPS component of gram-negative bacteria [26]. Therefore, in this study, we used LPS as an inflammatory agent to study the synergistic effects of carrageenan on the LPS-induced cellular inflammatory response. Furthermore, we developed a* Citrobacter freundii* (DBS100)-induced NIH mouse inflammation model to verify whether *κ*-carrageenan could aggravate bacterial-induced colitis in the mice intestinal tract. Our findings provide an evidence to support the conditional impact of carrageenan in vitro and in vivo.

## 2. Materials and Methods

### 2.1. Cell Culture and Stimulation with *κ*-Carrageenan and LPS

The human colonic carcinoma cell line HT-29 was purchased from the China Center for Type Culture Collection and was incubated with Eagle's Medium containing 10% fetal bovine serum at 37°C in an atmosphere with 5% CO_2_. Cells were cultured to 60–80% confluence and then treated with *κ*-carrageenan or LPS at the different dose and for the indicated time. The average molecular weight of 1 × 10^6^ of *κ*-carrageenan was used in this experiment. The *κ*-carrageenan was dissolved in ultrapure water at different concentrations (1, 10, 20, 40, and 60 *μ*g/mL) prior to experimentation.

### 2.2. Measurement of IL-8 Secretion by ELISA

HT-29 cells were treated with or without indicated dose of LPS (0.01, 0.1, and 1 *μ*g/mL) for 0.5 h, 1 h, 2 h, 4 h, 6 h, 12 h, and 24 h. The secretion of IL-8 was detected according to the ELISA kit (R&D Systems, Minneapolis, MN).

HT-29 cells were incubated with or without indicated dose of *κ*-carrageenan for 24 h or were pretreated with the different dose of *κ*-carrageenan for 1 h and then add LPS (1 *μ*g/mL) for 24 h. The secretion of IL-8 was detected according to the ELISA kit (R&D Systems, Minneapolis, MN).

### 2.3. Flow Cytometry Analysis

We use flow cytometry to measure the TLR4 expression of cell surface. HT-29 cells were incubated with the different dose of *κ*-carrageenan for 24 h or were pretreated with indicated dose of *κ*-carrageenan for 1 h and then LPS (1 *μ*g/mL) is added for incubating 24 h. The cells were collected and mixed on ice with 1 *μ*g PE-TLR4 antibody of against human. These cells were washed twice in PBS after 25 min and then examined by Beckman Gallios flow cytometer (Beckman Coulter, Inc., California, USA). The result was shown as mean fluorescence intensity.

We use flow cytometry to assess the LPS combined with HT-29 cells. HT-29 cells were incubated with the different dose of *κ*-carrageenan for 24 h and treated with LPS or *κ*-carrageenan for 1 h and then HT-29 cells were harvested with trypsin-EDTA. These cells were mixed with 1 *μ*g/mL FITC-LPS for 25 min and analyzed on a flow cytometer.

### 2.4. Real-Time Quantitative PCR (RT-qPCR)

For the in vitro analyses, we incubated HT-29 cells in the different dose of *κ*-carrageenan for 24 h or pretreated with indicated dose of *κ*-carrageenan for 1 h and then added them to LPS (1 *μ*g/mL) for 24 h. Then we harvested cells and isolated total RNA with RNAiso Plus Reagent (TaKaRa, Dalian, China). For the in vivo analyses, we extracted total RNA from gut mucosa using RNAiso Plus Reagent.

After RNA extraction, 0.4 *μ*g of total RNA was reverse-transcribed into cDNA in a 10 *μ*L reverse transcription reaction, and 2 *μ*L of the synthesized cDNA was used as template for PCR amplification. The primers used for the amplification of MD-2, CD14, and the reference gene, human*β-actin *(for in vitro experiments) or TLR4, NF-*κ*B, and the reference gene, mouse*β-actin* (for in vivo experiments) are listed in Table 1. Amplification reactions (20 *μ*L) included sample cDNA, primers and SYBR Premic Ex Taq II mix (TaKaRa, Dalian, China). RT-qPCR was conducted on the Mastercycler Ep Realplex real-time PCR system (Eppendorf, Hamburg, Germany) using SYBR-Green I in accordance with the manufacturer's instructions. The reaction mixtures were incubated at 95°C for 300 sec and then at 95°C for 10 sec, 60°C for 1 min, and 72°C for 15 sec for 40 cycles. The relative gene expression was determined by calculating the threshold cycle value, which was compared to the value for*β-actin* using the relative quantification method.

### 2.5. Western Blot Analysis

HT-29 cells were stimulated with the different concentrations of *κ*-carrageenan for 24 h or were pretreated with the indicated concentrations of *κ*-carrageenan for 1 h and then added to LPS (1 *μ*g/mL) for 24 h. Nuclear extracts were prepared using the NE-PER Nuclear and Cytoplasmic Extraction Kit (Pierce, Rockford, IL, USA) according to manufacturer's protocol. Protein concentrations were determined by the Bio-Rad DC Protein assay reagent.

Proteins (30 *μ*g) from nuclear extracts and whole cell lysates were separated by 10% sodium dodecylsulfate-polyacrylamide gel electrophoresis and were measured with antibodies of against TLR4, Bcl10, NF-*κ*B (CST, Danvers, MA, USA), or phospho-I*κ*B*α* (Abcam, Cambridge, UK), evaluated using the relevant HRP-linked mouse anti-human IgG (Abcam, Cambridge, UK). The immunoreactive proteins were visualized by enhanced chemiluminescence (ECL) kit (Santa Cruz Biotechnology, Santa Cruz, CA, USA). The results were quantified using NIH Image program by measuring the band intensity and compared to corresponding band intensity of *β*-actin.

### 2.6. Transient Transfection and Luciferase Reporter Assays

To measure the impact of *κ*-carrageenan on NF-*κ*B activation, we transiently cotransfected with Firefly luciferase reporter plasmid p-NF-*κ*B-Luc and Renilla luciferase plasmid p-RL into HT-29 cells and used X-treme GENE HP DNA Transfection Reagent for transfection. After transfection 10–12 h, cells were treated with *κ*-carrageenan for 24 h or treated with *κ*-carrageenan for 1 h and then stimulated with LPS (1 *μ*g/ml) for a further 24 h. The luciferase activities measurement of cell was used by a previously described method [22].

### 2.7. Experimental Animals

Male and female NIH (s) mice (3-week-old, weighing 15–20 g) were housed in pathogen-free conditions. All the mice were placed in clear plastic cages with free access to food and water, and each experimental group consisted of five males and five females. All experimental procedures were performed in accordance with the Guide for the Care and Use of Laboratory Animals of the National Institute of Health, and the Ethical Committee of Animal Use and Protection has approved animal protocols.

### 2.8. Infection and *κ*-Carrageenan Treatment

*κ*-Carrageenan was dissolved into saline. These indicated concentration (1.7 mg/kg, LOW; 8.3 mg/kg, MED; or 41.7 mg/kg, HIG) of *κ*-carrageenan were selected based on preliminary experiments in a related study [12, 27]. To measure the aggressive impact of *κ*-carrageenan on* C. freundii* DBS100-induced inflammation, *κ*-carrageenan was orally administered for 1 week prior to* C. freundii* DBS100 treatment.

The experimental model of inflammation was established using a previously described method with minor modifications [28]. The bacterial strain used in this study (DBS100,* Citrobacter freundii* biotype 4280) was purchased from the American Type Culture Collection (ATCC). Bacteria were grown at 37°C in Lysogeny broth (LB) (American Bioanalytical) or on MacConkey lactose agar plates. Water in the mouse cages was supplemented with streptomycin (5 mg/mL) for 48 h prior to bacterial inoculation. Infection was performed by oral gavage with 10^9^ CFU/mouse of* C. freundii* DBS100 grown overnight in LB broth (100 *μ*g/mL). Control mice were given same volume of sterile LB broth.

Animals were euthanized 10 days after inoculation. These symptoms of diarrhea, weight loss, and death were monitored after 10 days following the DBS100 administration. To determine the ratio of regulatory T cells (Tregs, CD4+CD25+CD127dim) and cytokine levels, blood and serum were collected from the mice through exsanguination by cardiac puncture. The severity of intestinal histopathology was graded and the entire colon was used to evaluate the severity of colonic tissues and expressions of NF-*κ*B, TLR4 at the mRNA level, whereas the paraformaldehyde-fixed (Merck, Darmstadt, Germany) or glutaraldehyde-fixed portion was used for further analyses, including histological examination, immunohistochemical analysis, and scanning electron microscopy (SEM).

80 mice were divided into eight groups, and each group have ten mice, including the blank group (sterile LB broth): the DBS100 group; the *κ*-carrageenan-groups (LOW; MED; HIG); and the *κ*-carrageenan + DBS100 groups (LOW + DBS100, MED + DBS100, and HIG + DBS100). Animals were euthanized 10 days after inoculation.

### 2.9. Macroscopic Evaluation

Table 2 details the criteria for macroscopic evaluation.

### 2.10. Histological Analysis

The colonic tissues were dissected, fixed with 4% paraformaldehyde, and then were paraffined and cut into 4 *μ*m thick; we used hematoxylin and eosin (H&E) to stain them. The histological grade and level of inflammation were blindly determined by veterinary pathologists. The colitis severity was quantified using the histological activity index (HAI) [29, 30], as previously described [31] and as detailed in Table 3.

### 2.11. Intestinal Mucosal Morphology

To identify morphology changes on the epithelial cell surface, intestinal segments were processed as previously described [32]. The segments were measured using Jeol JSM-6301 scanning electron microscope at ×200 magnification.

### 2.12. Examine of the CD4^+^CD25^+^CD127dim Ratio

100 *μ*L of peripheral blood was added to eFluor450, PE, APC, and eFluor710 labeled anti-mouse CD3, CD4, CD25, and CD127, respectively (all from eBioscience, California, USA). The mixture was incubated for 20 min. Then 1 mL of erythrocyte lysis solution was added for incubating 20 min. These cells were then centrifuged at 200 ×g for 5 min and added to 500 *μ*L PBS. Then we use flow cytometer to analyze samples.

### 2.13. Cytokine Analysis

The levels of proinflammatory cytokines, including interleukin 6 (IL-6), TNF-*α*, Th1 (IL-2, interferon IFN-*γ*), Th2 (IL-4, IL-10), and Th17 (IL-17) cytokines, were measured using the Cytokine Kit (BD BioSciences, NJ, USA). These serum samples and standards were measured by flow cytometer.

### 2.14. Immunohistochemical Analyses

For immunocytochemistry, colonic tissues were steeped with 5% paraformaldehyde and then were paraffined and cut into 4 *μ*m thick. Then anti-TLR4 antibody and anti-NF-*κ*B antibody (1 : 200, Abcam, Cambridge, UK) were used in this experiment. Nonimmunized mouse IgG at equal concentrations was used as a negative control for each primary antibody. Rabbit anti-rabbit IgG and goat anti-mouse IgG (1 : 200, ZSGB-BIO, Peking, China) were used as the secondary antibodies. Images were analyzed using Image Pro Plus 6.0 software (Media Cybernetics UK, Marlow, UK). The immunological histological chemistry (IHC) indexes for TLR4 and NF-*κ*B were determined as average integral optical densities according to the following equation: AIOD = (positive area/total area) × IOD.

### 2.15. Statistical Analysis

SPSS software, version 16.0 (SPSS Inc., Chicago, IL, USA), was used for statistical analyses. The data are shown as means ± SD. The differences of body weight between groups were analyzed through repeated measures ANOVA test. Macroscopic, histological score analyses were performed by student's* t*-test. One-way ANOVA after post hoc tests was used for others statistical analysis. *P* < 0.05 was considered as statistically significant.

## 3. Results

### 3.1. *κ*-Carrageenan Enhances LPS-Induced IL-8 Secretion in HT-29 Cells

**Figure 1:**
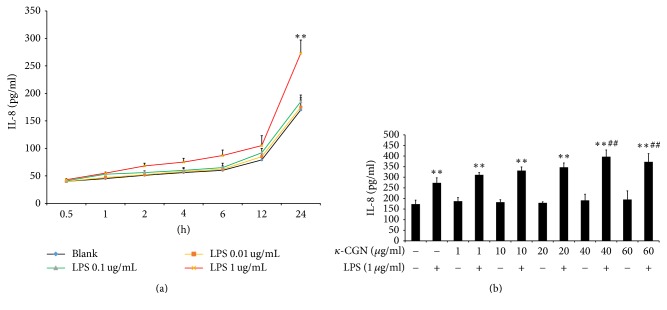
*κ*-Carrageenan enhances LPS-induced IL-8 secretion in HT-29 cells. (a) The effect of LPS on time-course and dose-response in HT-29 cells. (b) *κ*-Carrageenan increased LPS-induced expression of IL-8. IL-8 expression was assessed by ELISA in HT-29 cells treated with or without different concentrations of LPS (0.01, 0.1, and 1 *μ*g/mL) for 0.5 h, 1 h, 2 h, 4 h, 6 h, 12 h, and 24 h or treated with *κ*-carrageenan at the indicated doses in the absence or presence of 1 *μ*g/mL LPS. ^*∗∗*^ *P* < 0.01, compared with the blank group. ^##^ *P* < 0.01, compared with the LPS treated alone.

To determine the effect of LPS on time-course and dose-response, HT-29 cells were treated with or without different concentrations of LPS (0.01, 0.1, and 1 *μ*g/mL) for 0.5 h, 1 h, 2 h, 4 h, 6 h, 12 h, and 24 h. As shown in Figure 1(a), in 0.01 and 0.1 *μ*g/mL of LPS, the secretion of IL-8 experienced almost no change compared with the untreated sample (*P* > 0.05). However, HT-29 cells, treated with 1 *μ*g/mL LPS for 0.5–24 h, had approximately 7.5%–50.6% increase in the levels of IL-8 secretion as compared to untreated cells. The greatest marked effect was observed with 1 *μ*g/mL LPS (1.5-fold higher expression than untreated cells; *P* < 0.01) at 24 h. Our data showed that HT-29 cells treated with 0.01 and 0.1 *μ*g/mL LPS for 2–12 h were not effective. Therefore, in the present studies, at firstly we add different dose *κ*-carrageenan to HT-29 cells for 1 h and then add LPS for incubating 24 h.

To study whether*κ*-carrageenan may have differential effects depending on the inflammatory state of the cell, we treated HT-29 cells with*κ*-carrageenan in the presence and absence of LPS. As shown in Figure 1(b), in the absence of LPS, IL-8 production was not significantly different among the untreated sample and the samples treated with different doses of*κ*-carrageenan (*P* > 0.05). However, samples treated with 1 *μ*g/mL LPS had approximately 1.58-fold increase in the levels of IL-8 expression as compared to untreated cells (*P* < 0.01). Moreover, pretreatment with increasing concentrations of *κ*-carrageenan further increased the IL-8 levels induced by LPS. The greatest synergistic effect was observed with 1 *μ*g/mL LPS + 40 *μ*g/mL *κ*-carrageenan (1.45-fold higher expression than with LPS alone; *P* < 0.01). These results suggest that *κ*-carrageenan does not stimulate IL-8 on its own but enhances LPS-stimulated IL-8 expression in HT-29 cells.

### 3.2. *κ*-Carrageenan Pretreatment Enhances LPS-Induced Expression of TLR, MD-2, and CD14 but Does Not Promote LPS Combined with TLR4 in HT-29 Cells

**Figure 2:**
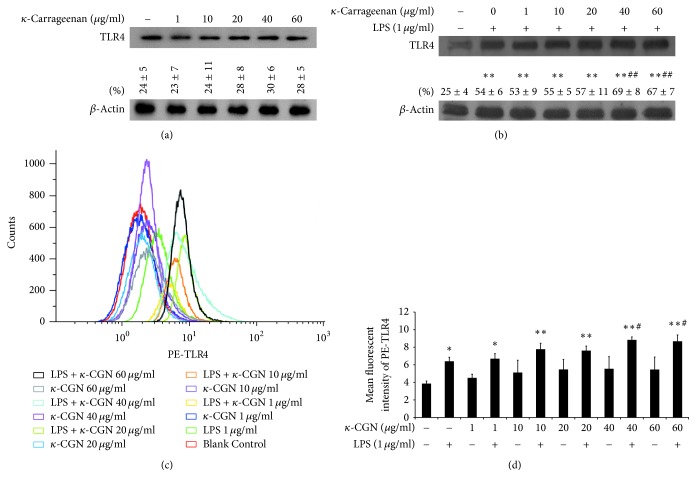
*κ*-Carrageenan enhances LPS-induced TLR4 expression in HT-29 cells. (a) The effect of *κ*-carrageenan on the expression of TLR4 in HT-29 cells. (b) *κ*-Carrageenan increased LPS-induced expression of TLR4. Cells were treated with *κ*-carrageenan for 24 h or were pretreated with *κ*-carrageenan for 1 h and then with LPS (1 *μ*g/mL) for 24 h. (c) TLR4 expression in HT-29 cells is affected by *κ*-carrageenan. Cells were treated with *κ*-carrageenan for 24 h and then were incubated with 1 *μ*g PE-TLR4 for 25 min. ^*∗*^ *P* < 0.05, ^*∗∗*^ *P* < 0.01, compared with the blank group. ^#^ *P* < 0.05, ^##^ *P* < 0.01 compared with those in the LPS alone treated group.compared with the LPS treated alone. (d) *y*-axis shows the mean fluorescent intensity of PE-TLR4.

To explore the mechanism by which *κ*-carrageenan increases LPS-dependent activation of IL-8, we assessed its effects on the expression and activity of TLR4, which is known to function as an inflammatory signaling receptor for LPS and other bacterial components. Treatment with increasing dose of *κ*-carrageenan did not significantly promote the protein expression of TLR4 as assessed by western blotting (Figure 2(a)). However, LPS markedly promoted the secretion of TLR4 (*P* < 0.01). Furthermore, pretreatment with 40 or 60 *μ*g/mL *κ*-carrageenan promoted additional enhancement of LPS-induced TLR4 expression (*P* < 0.01) (Figure 2(b)). In agreement with the western blot results, no significant changes in TLR4 expression could be detected by flow cytometry after treatment with *κ*-carrageenan alone, but TLR4 levels were increased by 54% after exposure to 1 *μ*g/mL LPS, with an additional increase observed for samples treated with *κ*-carrageenan + LPS (38 ± 8.18% additional increase for 40 *μ*g/mL + LPS; 35 ± 2.88% additional increase for 60 *μ*g/mL + LPS; *P* < 0.05) (Figures 2(c) and 2(d)).

**Figure 3:**
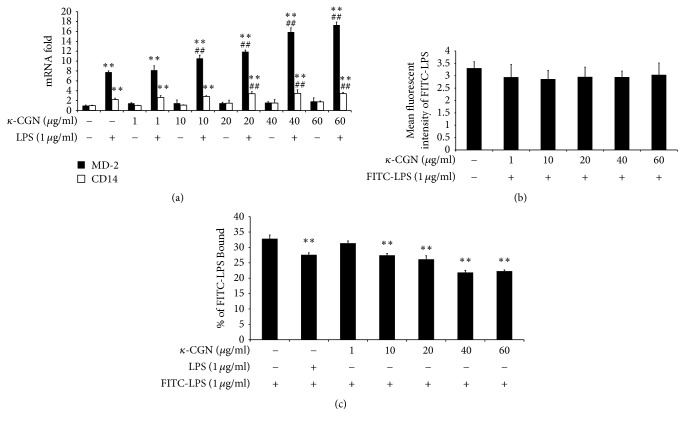
The impact of *κ*-carrageenan on mRNA expression of CD14 and MD-2 in LPS-induced HT-29 cells and binding of LPS. (a) The enhanced effect of *κ*-carrageenan on mRNA expression of CD14 and MD-2 in LPS-induced HT-29 cells. HT-29 cells were cultured with *κ*-carrageenan for 24 h or were pretreated with *κ*-carrageenan for 1 h and then stimulated with LPS (1 *μ*g/mL) for 24 h. (b) Effect of *κ*-carrageenan on FITC-LPS combined with cells. HT-29 cells were treated with the indicated concentrations of *κ*-carrageenan for 24 h and after cells were washed with PBS, and then 1 *μ*g/mL FITC-LPS was added for 25 min. The intensity of fluorescently labeled cells is shown as MFI of FITC-LPS. (c) *κ*-Carrageenan competitively blocks the binding of LPS. HT-29 cells were treated with 1 *μ*g/mL LPS or the indicated concentrations of *κ*-carrageenan for 1 h, and then 1 *μ*g/mL FITC-LPS was added for 25 min. ^*∗∗*^ *P* < 0.01, compared with the blank group. ^##^ *P* < 0.01 (*n* = 3), compared with the LPS treated alone.

While LPS triggers innate immune signaling by reciprocating with TLR4, LBP, MD-2, and CD14 are known to be required for the interaction between LPS and TLR4 [33–35]. Therefore, we performed RT-qPCR to determine whether *κ*-carrageenan may also modulate the expression of MD-2 and CD14. Our results showed that treatment with *κ*-carrageenan alone cannot significantly alter the expression of either gene. However, LPS alone significantly increased the expression of both genes (MD-2: ~8-fold increase; CD14: ~2-fold increase). Furthermore, pretreatment with *κ*-carrageenan before LPS promoted additional enhancement of the expression of both factors (2.3-fold increase for both genes, compared with LPS alone) (Figure 3(a)). These results suggest that *κ*-carrageenan synergizes with LPS to increase the expression of both MD-2 and CD14.

Because IL-8 is known to be activated by LPS signaling through TLR4, we considered the possibility that the increased levels of TLR4, MD-2, and CD14 could provide an explain for the effects of *κ*-carrageenan on IL-8 secretion. To measure whether the enhanced increase of TLR4, MD2, and CD14 through *κ*-carrageenan increases the binding of LPS to the cell surface, after 24 h treatment with *κ*-carrageenan, we applied FITC-LPS to HT-29 cells. *κ*-Carrageenan treatment for 24 h cannot upregulate FITC-LPS combined with the HT-29 cells (Figure 3(b)). As a potential explanation for these findings, we tested whether *κ*-carrageenan might also interplay with TLR4, therefore competitively inhibiting the connection with FITC-LPS. To determine this hypothesis, we use LPS or *κ*-carrageenan-treated cells for 1 h and then add FITC-LPS to the cells. Our results revealed that adding either 1 *μ*g/mL LPS or *κ*-carrageenan downregulated the bonding of FITC labeled LPS (Figure 3(c)), which indicates that *κ*-carrageenan binds to the same target as LPS. The decrease occurred in a concentration-dependent manner, which further verifies our results. Therefore, these experiments rule out the possibility that the upregulation of TLR4 or its signaling partners can explain the enhancement of IL-8 levels by *κ*-carrageenan in LPS-treated HT-29 cells.

### 3.3. *κ*-Carrageenan Enhances LPS-Stimulated Activation of NF-*κ*B

**Figure 4:**
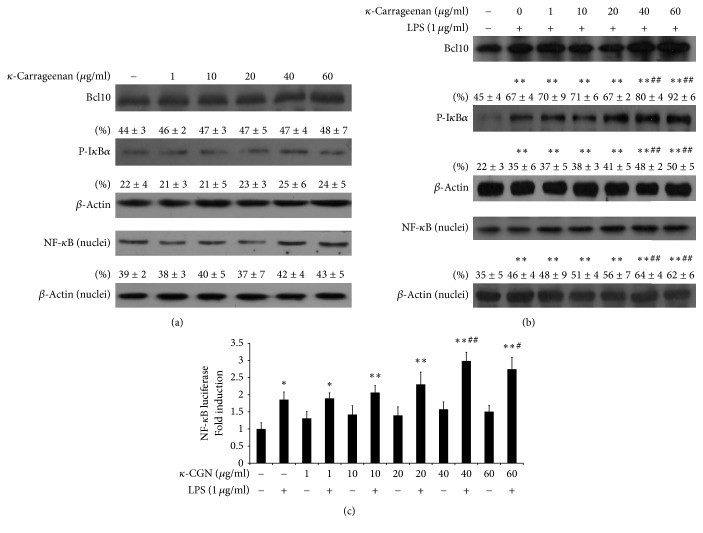
*κ*-Carrageenan potentiates the LPS-stimulated activation of NF-*κ*B. (a) The effect of *κ*-carrageenan on the expression of Bcl10, NF-*κ*B, and p-I*κ*B*α* in HT-29 cells. (b) *κ*-Carrageenan enhances the LPS-stimulated secretion of Bcl10, NF-*κ*B, and p-I*κ*B*α*. HT-29 cells were treated with *κ*-carrageenan for 1 h. (c) *κ*-Carrageenan induces transcriptional activation of NF-*κ*B. ^*∗*^ *P* < 0.05, ^*∗∗*^ *P* < 0.01, compared with the blank group. ^#^ *P* < 0.05, ^##^ *P* < 0.01, compared with the LPS treated alone.

Because *κ*-carrageenan failed to promote the binding of LPS to TLR4, we considered other possible explanations for its ability to cooperate with LPS to increase the IL-8 secretion. To determine whether *κ*-carrageenan could enhance Bcl10-mediated LPS-induced activation in HT-29 cells, we performed western blotting assays. As shown in Figure 4(a), *κ*-carrageenan alone mediated a slight nonstatistical increase in the expression of Bcl10, the phosphorylation of I*κ*B*α*, and the nuclear expression of NF-*κ*B in HT-29 cells at high doses (*P* > 0.05). Additionally, 1 *μ*g/mL LPS increased Bcl10 expression by 48.89% and I*κ*B*α* phosphorylation by 59.10% (*P* < 0.01). Furthermore, *κ*-carrageenan pretreatment markedly enhanced the LPS-dependent increase in Bcl10 expression and I*κ*B*α* phosphorylation in a dose-dependent manner (*P* < 0.01, Figure 4(b)). The expression of NF-*κ*B was also enhanced significantly at lower *κ*-carrageenan concentrations (40–60 *μ*g/mL; *P* < 0.01).

To confirm the induction of NF-*κ*B transcriptional activity by *κ*-carrageenan and LPS, we performed luciferase assays using a NF-*κ*B reporter plasmid. Consistent with our other findings, *κ*-carrageenan treatment induced a low-level increase in the transcription activity of NF-*κ*B, but the increase was not statistically significant. However, treatment with LPS significantly increased the transcriptional activity of NF-*κ*B (*P* < 0.01), and pretreatment with *κ*-carrageenan potentiated this LPS-stimulated transcriptional activity of NF-*κ*B in HT-29 cells. These findings are coincident with previously published results suggesting that *κ*-carrageenan potentiated LPS-stimulated activation of NF-*κ*B mediated by Bcl10 [18].

### 3.4. Administration of *κ*-Carrageenan Potentiates DBS100-Induced Weight Loss and Mortality in a Mouse Inflammatory Model

**Figure 5:**
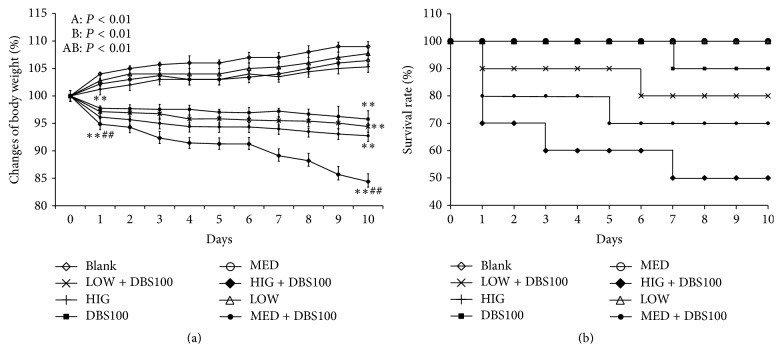
The impact of *κ*-carrageenan on weight loss and the mortality with* Citrobacter freundii* DBS100-stimulated inflammation. (a) Weight changes in percentage. (b) Survival ratios. *κ*-Carrageenan was orally administered for 1 week prior to bacterial inoculation. Infection was performed by oral gavage with 10^9^ CFU/mouse of* C. freundii* DBS100. After ten days, the weight and the survival rate of the animals were recorded. Repeated measures ANOVA was used to measure differences between the groups for A; differences over time for B and differences between the interaction of experiment and time for AB shown. ANOVA and LSD post hoc test were used to examine pairwise comparisons between the groups at individual time points. ^*∗∗*^ *P* < 0.01, compared with the blank group. ^##^ *P* < 0.01, compared with the* C. freundii* DBS100-treated group.

LPS is an important constituent of the external wall of gram-negative bacteria, and lots of the inflammatory signals were triggered through LPS component of gram-negative bacteria [26]. Based on our in vitro findings, we postulated that the inflammatory response induced in vivo by a gram-negative bacterium might also be enhanced by *κ*-carrageenan. To explore this possibility, we established a* C. freundii *DBS100-induced inflammation model in mice. The effect of *κ*-carrageenan pretreatment on* C. freundii* DBS100-induced gut damage was investigated by measuring survival rate and weight loss. As shown in Figure 5(a), any comparisons between the groups had marked differences, other than *κ*-carrageenan alone-treated mice (LOW, MED, and HIGH) on days 1 to 10 (*P* < 0.01). However, the body weights of the* C. freundii* DBS100-treated mice were markedly decreased at day 1, with a more significant difference at day 10 (*P* < 0.01); in addition, the *κ*-carrageenan + DBS100-treated mice had significantly greater decreases in body weight than the DBS100 mice. The HIG + DBS100 group showed the most dramatic result, with the body weight decreasing by 5.1%  ± 1.01 (for DBS100 mice) and by 15.7%  ± 1.43 (for HIG + DBS100 mice) at day 10 (*P* < 0.01).

At the end of the experiment (day 10), no mice in the blank or the *κ*-carrageenan groups died; however, one mouse in the* C. freundii* DBS100-treated group died (1/10) at day 7 (Figure 5(b)). Additionally, treatment with *κ*-carrageenan prior to bacterial infection obviously increased the number of dead mice, even starting at day 1; there were 1, 2, and 3 dead mice in the LOW + DBS100, MED + DBS100, and HIG + DBS100 groups. By day 10, the number of dead mice in the HIG + DBS group reached 5 (i.e., 50% mortality). Thus, these findings demonstrate the exacerbatory effect of *κ*-carrageenan towards bacterial-induced inflammatory gut disease in the mouse.

### 3.5. Effects of *κ*-Carrageenan on DBS100-Induced Colon Damage

**Figure 6:**
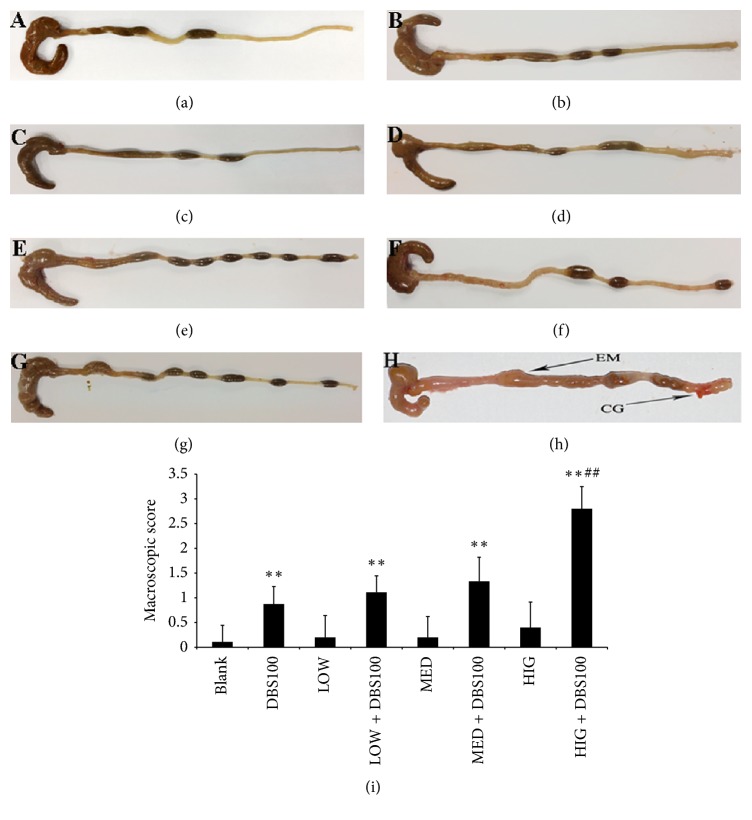
The impact of *κ*-carrageenan on colon damage following* Citrobacter freundii* DBS100. (a) Blank group; (b) DBS100 treated alone; (c) LOW treated alone; (d) LOW + DBS100 cooperative accessing; (e) MED treated alone; (f) MED + DBS100 cooperative accessing; (g) HIG treated alone; (h) HIG + DBS100 cooperative accessing; (i) colon damage scores at 10 days after DBS100 administration. ^*∗∗*^ *P* < 0.01, compared with the blank group. ^##^ *P* < 0.01, compared with the* C. freundii* DBS100-treated group. CG, congestion; EM, edema.

To further explore the in vivo effects of *κ*-carrageenan in exacerbating* C. freundii* DBS100-induced gut inflammation, we examined the intestinal mucosal gut morphology of the eight groups of mice. The symptoms of inflammation were obvious after exposure to* C. freundii* DBS100 for 10 days (Figure 6, see (b) versus (a)). The pathologic changes included a slight increase in the rigidity of the colon and signs of colonic thickening, which are consistent with previously reported symptoms for the entire colon after exposure to* C. freundii* DBS100 [28].

Furthermore, *κ*-carrageenan treatment alone had no detectable impact on the colons of the LOW, MED, and HIG groups of animals, which exhibited apparently normal colonic mucosa (Figures 6(c), 6(e), and 6(g)). However, pretreatment of high-dose *κ*-carrageenan in the HIG + DBS100 group led to more severe inflammation (Figure 6(h)). This group of mice showed invasive hyperemia, bleeding ulceration, and focal mucosal necrosis. The macroscopic inflammation scores for colon damage supported our observations (Figure 6(i)). Therefore, our results verify that *κ*-carrageenan potentiates* C. freundii* DBS100-stimulated colitis.

### 3.6. Impact of *κ*-Carrageenan on the Histology of DBS100-Stimulated Colon Sections

**Figure 7:**
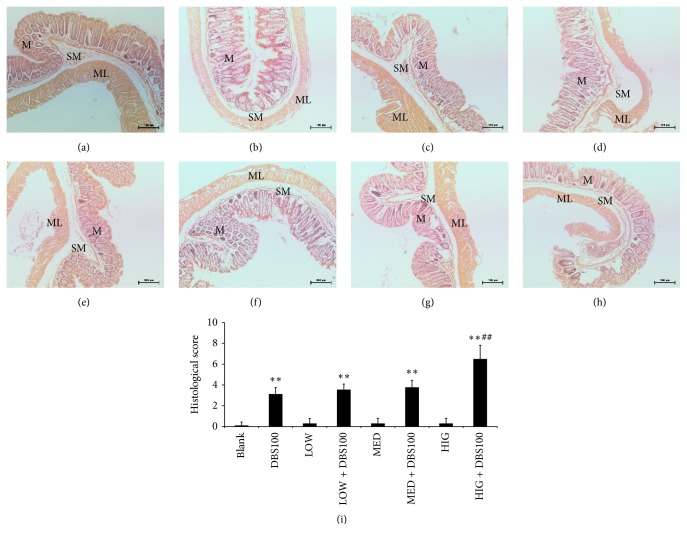
The impact of *κ*-carrageenan on histological changes and the HAI scores of* Citrobacter freundii* DBS100-stimulated mice. (a) Blank group; (b) DBS100 treated alone; (c) LOW treated alone; (d) LOW + DBS100 cooperative accessing; (e) MED treated alone; (f) MED + DBS100 cooperative accessing; (g) HIG treated alone; (h) HIG + DBS100 cooperative accessing. H&E: ×200. (i) The HAI scores of colonic in* C. freundii* DBS100-stimulated mice. ^*∗∗*^ *P* < 0.01, compared with the blank group; ^##^ *P* < 0.01, compared with the DBS100-treated group. M, mucosa; SM, submucosa; ML, muscle layer.

To further examine the effects of *κ*-carrageenan on* C. freundii* DBS100-induced gut inflammation at the animal level, we performed histological analysis of gut tissue sections (Figure 7). The blank group indicated regular architecture, with abundant of goblet cells and an unbroken epithelial layer and crypts (a). However, compared with the blank group, colonic sections from DBS100-infected mice indicated crypt elongation, goblet cell loss, and mild inflammation ((b) versus (a)). Similar to the blank group, the colonic tissues of the *κ*-carrageenan-treated groups also showed no obvious inflammatory presentations ((c), (e), and (g)). However, *κ*-carrageenan aggravated the microscopic colon damage induced by DBS100 for the HIG + DBS100 group (h). This group possessed symptoms of architectural crypt distortion, epithelial damage, mucosal inflammation with inflammatory cell infiltration in the lamina propria, reduced numbers of goblet cells, and submucosa edema. These results were supported by the HAI scores, which were 2.08-fold higher for the HIG + DBS100 group than for the DBS100 group (*P* < 0.01; Figure 7(i)).

**Figure 8:**
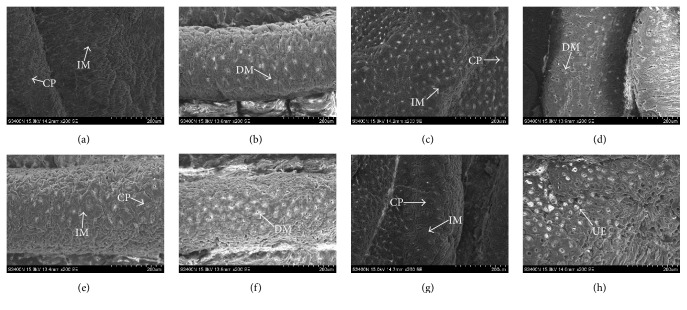
SEM of morphology changes. (a) Blank group; (b) DBS100 treated alone; (c) LOW treated alone; (d) LOW + DBS100 cooperative accessing; (e) MED treated alone; (f) MED + DBS100 cooperative accessing; (g) HIG treated alone; (h) HIG + DBS100 cooperative accessing, ×200-fold. CP, crypts; IM, intact microvilli; DM, disintegrating microvilli; MS, mucus secretion at crypt opening; UE, ulcers and empty.

To verify these findings, we performed scanning electron microscopy (SEM) (Figure 8). The blank group exhibited a normal appearance, with intact microvilli and normal epithelial cell surface and crypt architecture (a). However, the colons from the DBS100 group had injury to the mucosa skin and effacement of the microvilli (b). *κ*-Carrageenan treatment cannot impact the morphology in the absence of* C. freundii* DBS100 ((c), (e), and (g)); however, for mice infected with bacteria, *κ*-carrageenan exacerbated the morphological variation of the colon in a concentration-dependent manner ((d), (f), and (h)). There was more pronounced injury and diffuse ulcers on the mucosa surface in the HIG + DBS100 group (h). Collectively, these results verify at both the macroscopic and the microscopic levels that *κ*-carrageenan aggravates* C. freundii* DBS100-induced gut inflammation.

### 3.7. *κ*-Carrageenan Administration Exacerbates the Reduction in Tregs by* C. freundii* DBS100

**Figure 9:**
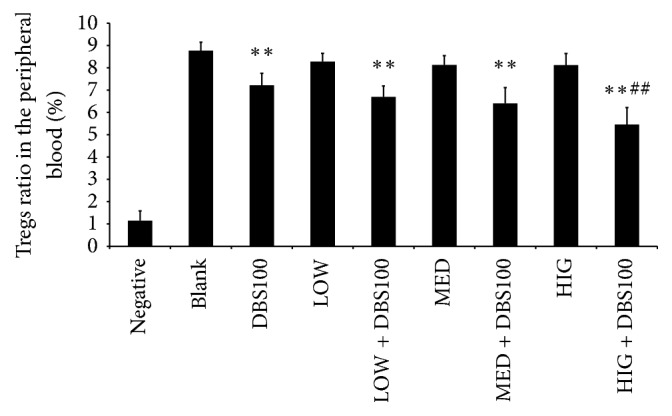
Ratio of CD4+CD25+CD127dim/CD4+ (Tregs) in mice. ^*∗∗*^ *P* < 0.01, compared with the blank group. ^##^ *P* < 0.01, compared with the* Citrobacter freundii* DBS100 treated alone group.

Tregs are subpopulation of T cells that reduce inflammatory processes by maintaining tolerance to self-antigens [36]. To examine the impact of *κ*-carrageenan on the Treg subpopulation in mice treated with or without* C. freundii*, we performed flow cytometric analysis (Figure 9). Our results show that the ratio of Tregs in systemic circulation was markedly reduced in the DBS100 group compared to the blank group (7.21%  ± 0.54 versus 8.77%  ± 0.38; *P* < 0.01). Furthermore, *κ*-carrageenan alone did not affect the Treg ratio, but pretreatment with *κ*-carrageenan promoted further decrease in DBS100-infected mice. The decrease was the greatest in the peripheral blood from the HIG + DBS100, which showed a 24.17% reduction relative to the DBS100 group. These findings demonstrate that *κ*-carrageenan inhibits the activity of regulatory T cells, which may further aggravate the imbalance of the immune response.

### 3.8. The Impact of *κ*-Carrageenan Administration on Systemic Cytokine Levels in DBS100-Induced Colonic Inflammation

We assessed the levels of the proinflammatory (TNF, IL-6), Th1 (IFN-*γ* and IL-2), Th2 (IL-4, IL-10), and Th17 (IL-17A) cytokines in serum samples of mice (Table 4). The levels of TNF, IL-6, IFN-*γ*, IL-2, and IL-17A were elevated in the DBS100 group compared with blank group (*P* < 0.05 or *P* < 0.01); in contrast, the levels of IL-10, which mediates anti-inflammatory T cell responses, were significantly decreased (*P* < 0.05), while IL-4 levels were below the detection limit.

Some slight increases in the cytokine levels were observed in the serum of *κ*-carrageenan alone groups, but these increases were not significant (*P* > 0.05). However, the *κ*-carrageenan + DBS100 groups tended to have higher levels of cytokines than the DBS100 group. This was especially noted for the HIG + DBS100 group compared to the* C. freundii* DBS100 group (*P* < 0.05). On the other hand, the administration of high-dose *κ*-carrageenan led to markedly collaborative downregulation for the IL-10 levels (4.61 ± 0.671 pg/mL versus 8.95 ± 1.01 pg/mL for the DBS100 group; *P* < 0.05).

### 3.9. Impact of *κ*-Carrageenan on TLR4 and NF-*κ*B Secretion in the Gut Mucosa

**Figure 10:**
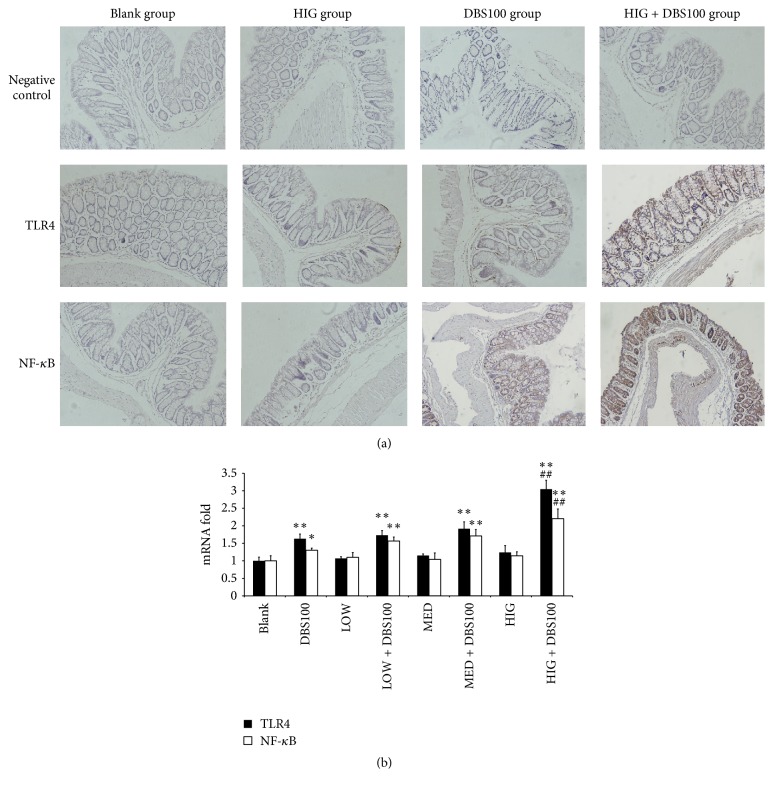
The impact of *κ*-carrageenan on the secretion of TLR4 and NF-*κ*B in the colonic mucosa of experimental mice. (a) The effect of *κ*-carrageenan on the secretion of TLR4 and NF-*κ*B in mice, ×200 fold. (b) The impact of *κ*-carrageenan on the expression of TLR4 and NF-*κ*B at the mRNA level. ^*∗*^ *P* < 0.05, ^*∗∗*^ *P* < 0.01, compared with the blank group. ^##^ *P* < 0.01, compared with the* Citrobacter freundii* DBS100-treated group.

To determine whether in the inflammatory mouse model, like in HT-29 cells, *κ*-carrageenan enhances inflammation-dependent secretion of TLR4 and NF-*κ*B, we examined the effects of *κ*-carrageenan on TLR4 and NF-*κ*B secretion in the gut mucosa. The secretion of TLR4 and NF-*κ*B in the colonic epithelium was induced by* C. freundii* DBS100 (*P* < 0.01). Induction was not observed for *κ*-carrageenan alone, but *κ*-carrageenan caused further upregulation in mice treated with* C. freundii* DBS100 in a concentration-dependent manner (Figure 10(a), Table 5). The most significant upregulation was observed in the HIG + DBS100 group, which showed a 1.29-fold upregulation in TLR4 levels and a 1.32-fold upregulation in NF-*κ*B levels compared with the DBS100 group (*P* < 0.01).

We also examined the expression of TLR4 and NF-*κ*B at the mRNA level by quantitative RT-PCR. As shown in Figure 10(b), a similar pattern of regulation was observed for these mRNAs. These results verify that *κ*-carrageenan enhances the* C. freundii* DBS100-dependent induction of TLR4 and NF-*κ*B in the intestinal mucosa of infected mice.

## 4. Discussion

An important role of the intestinal tract is to absorb nutrients while resisting entry of invasive pathogens that are involved in the inflammatory response. When the intestinal tract is infected by pathogens, microbe-associated molecular patterns, such as LPS, upregulate the level of proinflammatory cytokines in the colon through classical inflammatory signaling pathways [37–39]. Considering the controversy surrounding the safety of carrageenan and its inflammatory properties in animal models, the present study focused on the potential ability of *κ*-carrageenan to enhance LPS-mediated inflammatory pathway in human colonic cells. To achieve this purpose, we used a* C. freundii* DBS100-induced intestinal inflammation model to determine the impact of *κ*-carrageenan on aggravate inflammatory of the intestinal tract.

A previous study demonstrated the impact of carrageenan on the production of IL-8 in the human colonic epithelial cell line NCM460. Treatment with 1 *μ*g/mL *λ*-, *κ*-, or ι-carrageenan was shown to result in 2.03-fold, 1.59-fold, or 1.44-fold increase in IL-8 secretion by NCM460 cells [19]. In the present study, we observed only about 1.12-fold increase in IL-8 secretion in HT-29 cells after treatment with 60 *μ*g/mL *κ*-carrageenan alone. This difference between the two studies may relate to differences in the degree of differentiation of the colon epithelial cells that were used. Some reports have shown that carrageenan could induce cancer and ulcer of gut [40, 41]. Epithelial cells are the first line of defense of the intestinal mucosa. Their collapse resulted in the aggregation of inflammatory cells [42]. Although NCM460 comes from the normal human intestinal epithelial cells, it was transformed minimally to be an immortalized cell stain, and it can express TLR4 by itself [43]; whereas HT-29 is moderately differentiated colon epithelial cell, the expression of TLR4 is very low in HT-29 cell [44]. In our research, TLR4 is one of the targets of carrageenan and LPS. The low secretion of TLR4 in HT-29 cells will be a better cell model to research the synergy impact of carrageenan on LPS-stimulated expression and activity of TLR4. HT-29 cells also express lots of proinflammatory cytokines including interferon-*γ*, TNF-*α*, and IL-1, which will induce the release of other inflammatory mediators, such as IL-8, which are increased in IBD [45, 46]. In this research, it is obvious that the amount of IL-8 induced with *κ*-carrageenan alone is not enough to activate an inflammatory response. However, a common finding of our study is that *κ*-carrageenan was found to synergize with LPS to trigger an enhanced inflammatory response, though the extent of the synergy appears to be high for HT-29 cells. Pretreatment with carrageenan also has been demonstrated to enhance LPS-induced TNF-*α* production in whole blood from mice [47]. These results indicate that carrageenan enhances LPS-induced inflammation. Thus, *κ*-carrageenan may either increase the signaling within the LPS-induced inflammatory signaling pathway or provide a parallel pathway with a similar end result of enhancing inflammatory response.

LPS can recognize TLR4. When LPS interacts with TLR4, a cascade is activated that can lead to systemic inflammatory response [20, 48, 49]. We demonstrated that *κ*-carrageenan alone can mediate a slight increase in the secretion of TLR4 in HT-29 cells and that LPS can mediate a significant increase but that the levels were the greatest after treatment with both LPS and *κ*-carrageenan. Furthermore, the levels of the auxiliary proteins MD-2 and CD14, which are needed for TLR4 signaling, were synergistically increased. Thus, our data seems to be consistent with a model in which *κ*-carrageenan enhances LPS-induced secretion of IL-8 by enhancing TLR4 signaling. However, *κ*-carrageenan pretreatment cannot promote the cell surface FITC-LPS combined with TLR4 and in fact functioned as a competitive inhibitor of LPS, which is consistent with the demonstration that *κ*-carrageenan can bind TLR4 [50] and suggests that the synergy may occur at other targets in the signaling pathway.

We assessed the activation of the Bcl10-NF-*κ*B inflammatory pathway in HT-29 cells. Our data indicated that treatment of HT-29 with LPS significantly increases Bcl10, phospho-I*κ*B*α*, and NF-*κ*B expression, which is consistent with previous studies. Bcl10 is known to be a potential mediator of LPS-induced activation of NF-*κ*B [20]. Additionally, *λ*-carrageenan mediates prolonged activation of both Bcl10 and NF-*κ*B in HT-29 and NCM460 cells [48, 50]. However, we found that high doses of *κ*-carrageenan alone could only mediate a slight, nonstatistical upregulation of the expression of Bcl10, phosphorylated I*κ*B*α*, and NF-*κ*B. Interestingly, when *κ*-carrageenan was applied before LPS treatment, the expression of Bcl10, phosphorylation of I*κ*B*α*, nuclear level of p65 subunit, and transcription activity of NF-*κ*B were significantly increased. These results clearly demonstrated that *κ*-carrageenan enhances LPS-induced secretion of IL-8 not through enhancing TLR4 signaling, because *κ*-carrageenan cannot promote the cell surface FITC-LPS combined with TLR4 and suggests that *κ*-carrageenan can competitively inhibit LPS combining with TLR4 receptor. However, *κ*-carrageenan enhances LPS-induced IL-8 secretion by stimulating the Bcl10-NF-*κ*B pathway in HT-29 cells.

Given that LPS is derived from gram-negative bacteria, we also asked whether *κ*-carrageenan could promote the inflammatory response induced by a gram-negative pathogen in vivo. To answer this question,* C. freundii *DBS100 was used to establish an intestinal inflammation model, because its virulence factors are similar to those of enteropathogenic* Escherichia coli *of human [28, 51]. Furthermore,* C. freundii *DBS100 induces inflammatory processes that are similar to those of inflammatory bowel disease in humans [28]. Our data show no weight loss, no mortality, and similar histological appearance as in untreated mice for the *κ*-carrageenan alone treatment groups, which indicates that *κ*-carrageenan alone does not cause inflammation in vivo. This conclusion is consistent with studies demonstrating that carrageenan does not produce intestinal ulcerations at doses of up to 5% [52]. Carrageenan has also been demonstrated not to be carcinogenic in long-term bioassays in rodents [4]. Our results showed that administration of* C. freundii* DBS100 resulted in a minimal weight loss and histological damage, with 10% mortality, which is close to the values from the literature [28]. However, *κ*-carrageenan pretreatment before* C. freundii* DBS100 infection resulted in significantly weight loss, with serious colon damage and 20–50% mortality, depending on the *κ*-carrageenan dose. Morphological and histological data were consistent with the possibility that intragastric administration of *κ*-carrageenan can aggravate the* C. freundii* DBS100-induced inflammatory death of mice.

Previous studies have addressed the ability of carrageenan to increase the proinflammatory cytokines TNF, IL-8, and IL-6 [19, 20, 53]. In our study, IL-6 and TNF were not activated by *κ*-carrageenan alone but were significantly increased by *κ*-carrageenan in conjunction with* C. freundii*. We also examined the upregulation of cytokines produced by Th1 cells, including IFN-*γ* and IL-2 [28], and observed an analagous pattern of regulation. Several reports have demonstrated that IL-17A is a major driver of intestinal inflammation [54] through its ability to activate T cell production [55]. Consistently, *κ*-carrageenan provided a similar synergistic increase in the level of IL-17A in* C. freundii *DBS100-treated mice. Thus, our findings support the ability of *κ*-carrageenan to conditionally exacerbate the inflammatory response.

Tregs are important for maintaining homeostasis in the intestine and controlling inflammation by inhibiting the activation and proliferation of CD4^+^ and CD8^+^ cells [36]. In this study, we found that *κ*-carrageenan did not significantly affect Treg levels on its own but significantly reduced the ratio of Tregs in a dose-dependent manner in mice exposed to* C. freundii *DBS100, which is consistent with our other findings and suggests a mechanism of humoral immune tolerance activated by *κ*-carrageenan under inflammatory environmental conditions.

Finally, we examined the* C. freundii *DBS100 inflammatory model to determine whether the in vivo exacerbatory effects of *κ*-carrageenan are mediated through the Bcl10-NF-*κ*B signaling pathway. The different doses of *κ*-carrageenan pretreated mice indicated a marked upregulation in the DBS100-stimulated secretion of NF-*κ*B, indicating that NF-*κ*B might constitute a key component in the *κ*-carrageenan intestinal inflammatory exacerbation. TLR4 was also increased; however, whether increased TLR4 plays a role in the inflammation requires further investigation.

In conclusion, we demonstrated enhanced effects of *κ*-carrageenan on the inflammatory reaction in vitro and in vivo. *κ*-Carrageenan increased the expression of TLR4 receptor but competitively bound TLR4, blocking LPS binding, which rules out the enhanced TLR4 levels as an explanation for the synergistic effects of *κ*-carrageenan. However, our results also showed that *κ*-carrageenan can participate in the Bcl10-NF-*κ*B-mediated pathway to enhance LPS stimulated secretion of IL-8 in HT-29 cells. The* C. freundii* DBS100-induced intestinal inflammation model further verified that *κ*-carrageenan could aggravate the inflammatory reaction of the colon to pathogen exposure *κ*-carrageenan modulated cytokine production, downregulated the proportion of Tregs, and upregulated NF-*κ*B, all of which are likely to contribute to the exacerbatory effects of *κ*-carrageenan. Collectively, our results suggest that *κ*-carrageenan serves as a potential inflammatory agent that magnifies existing intestinal inflammation.

## Figures and Tables

**Table 1:** Oligonucleotide primers used in this work.

Primers	Primers (5′—3′)	Population information	Gene ID
MD-2-F	AAAGAAGTTATTTGCCGAGGAT	Human	AB018549.1
MD-2-R	TTCCCTTGAAGGAGAATGATAT	Human	
CD14-F	TCTGACAATCCTGGACTGGG	Human	M86511.1
CD14-R	GTGGGCGTCTCCATTCCT	Human	
*β*-Actin-F	TGGAATCCTGTGGCATCCATGAAAC	Human	NM_001101.3
*β*-Actin-R	TAAAACGCAGCACAGTAACAGTCCG	Human	
TLR4-F	GCTGCAACTGATGTTCCTTCT	Mouse	NM_021297.3
TLR4-R	CCCAACATTCATCCATCTCA	Mouse	
NF-*κ*B-F	AAAGCCCTGACAGTCCATTG	Mouse	NM_008689.2
NF-*κ*B-R	TTGCTAGACACCGTCTGTGC	Mouse	
*β*-Actin-F	TTGCTGACAGGATGCAGAAG	Mouse	NM_007393.5
*β*-Actin-R	ACATCTGCTGGAAGGTGGAC	Mouse	

**Table 2:** Macroscopic evaluation of colonic tissues.

Score	Histological changes	Length of bowel damage
0	Normal	None
1	Erythema	<20%
2	Slight dropsy, and small erosions	21–40%
3	More hemorrhagic ulcers or moderate inflammation or conglutination	41–60%
4	Severe ulceration and serious conglutination	>61%

**Table 3:** The histologic activity index (HAI) scoring system for inflammation-associated histological changes in the colon.

Score	Infiltrate	Extent of inflammatory infiltrate	Goblet cell mucin depletion	Nature of mucosal changes
0	Normal/physiologic	None	None	None
1	Minimal elevation	Single or rare, scattered foci	Minimal	Minimal deterioration
2	Expanded within or beyond lamina propria	Patchy, moderately abundant	Moderate	More deterioration
3	Crypt abscesses or submucosal involvement	Extensive	Extensive	More thanatosis
4	Diffuse	Inflammatory cells in the gut lumen	N/A	N/A

**Table 4:** Expression levels of the inflammatory cytokines (pg/ml, mean ± SD).

Group	IL-10	IL-17A	TNF	IFN-*γ*	IL-6	IL-4	IL-2
Blank	13.32 ± 1.83	1.96 ± 0.20	8.94 ± 0.72	2.45 ± 0.41	5.32 ± 1.68	5.74 ± 1.27	2.27 ± 1.83
DBS100	8.95 ± 1.01^*∗*^	3.93 ± 0.58^*∗*^	14.25 ± 2.74^*∗*^	7.57 ± 1.92^*∗*^	19.12 ± 1.98^*∗∗*^	≦0	6.65 ± 2.5^*∗∗*^
LOW	12.37 ± 1.75	2.17 ± 1.28	8.07 ± 0.82	2.13 ± 0.27	5.67 ± 1.37	≦0	3.2 ± 2.12
LOW + DBS100	8.07 ± 1.00^*∗∗*^	4.18 ± 0.76^*∗*^	15.21 ± 1.59^*∗∗*^	7.90 ± 2.80^*∗*^	21.36 ± 4.54^*∗∗*^	4.13 ± 2.02	8.78 ± 1.75^*∗∗*^
MED	12.53 ± 1.89	2.12 ± 0.52	9.35 ± 0.78	2.41 ± 0.19	5.52 ± 1.24	≦0	2.68 ± 1.62
MED + DBS100	6.45 ± 1.55^*∗∗*^	4.65 ± 0.36^*∗∗*^	16.17 ± 2.75^*∗∗*^	11.50 ± 1.69^*∗∗*^	26.43 ± 3.84^*∗∗*^	3.49 ± 1.67	8.16 ± 3.04^*∗∗*^
HIG	11.44 ± 1.70	2.31 ± 0.61	9.11 ± 2.13	2.28 ± 0.36	5.53 ± 1.73	≦0	2.21 ± 2.44
HIG + DBS100	4.61 ± 0.67^*∗∗*#^	5.97 ± 1.47^*∗∗*#^	20.96 ± 1.76^*∗∗*##^	14.56 ± 1.67^*∗∗*##^	34.06 ± 5.26^*∗∗*##^	1.54 ± 1.32	12.49 ± 2.66^*∗∗*##^

Notes: ^*∗*^ *P* < 0.05, compared with the blank group. ^*∗∗*^ *P* < 0.01, compared with the blank group. ^#^ *P* < 0.05, compared with the DBS100 treated alone group. ^##^ *P* < 0.01, compared with the DBS100 treated alone group.

**Table 5:** The effect of carrageenan on the secretion of TLR4 and NF-*κ*B (Au, mean ± SD).

Group	Negative control	TLR4	NF-*κ*B
Blank	58.64 ± 5.21	345.61 ± 13.36	314.47 ± 13.39
DBS100	29.72 ± 6.80	425.59 ± 15.91^*∗∗*^	394.03 ± 15.85^*∗∗*^
LOW	65.15 ± 5.51	337.40 ± 23.34	319.07 ± 11.97
LOW + DBS100	67.84 ± 3.49	440.35 ± 10.73^*∗∗*^	415.03 ± 15.66^*∗∗*^
MED	65.95 ± 10.35	353.72 ± 10.54	328.22 ± 23.03
MED + DBS100	61.33 ± 8.42	472.67 ± 18.17^*∗∗*^	433.01 ± 25.87^*∗∗*^
HIG	86.11 ± 24.69	379.90 ± 8.19	339.67 ± 10.76
HIG + DBS100	47.78 ± 7.78	546.71 ± 16.97^*∗∗*##^	520.03 ± 17.15^*∗∗*##^

Notes: ^*∗∗*^ *P* < 0.01, compared with the blank group; ^##^ *P* < 0.01, compared with the DBS100 treated alone group.
